# Implementation of early prophylaxis for deep-vein thrombosis in intracerebral hemorrhage patients: an observational study from the Chinese Stroke Center Alliance

**DOI:** 10.1186/s12959-024-00592-w

**Published:** 2024-02-28

**Authors:** Ran Zhang, Weige Sun, Yana Xing, Yongjun Wang, Zixiao Li, Liping Liu, Hongqiu Gu, Kaixuan Yang, Xin Yang, Chunjuan Wang, Qingbo Liu, Qian Xiao, Weixin Cai

**Affiliations:** 1 Nursing Department, Beijing Tiantan Hospital, Capital Medical University, No.119 South Fourth Ring West Road, Fengtai District, 100070 Beijing, China; 2 Department of Neurology, Beijing Tiantan Hospital, Capital Medical University, Beijing, China; 3 China National Clinical Research Center for Neurological Diseases, Beijing, China; 4 Center of Stroke, Beijing Institute for Brain Disorders, Beijing, China; 5 Beijing Key Laboratory of Translational Medicine for Cerebrovascular Disease, Beijing, China; 6 National Center for Healthcare Quality Management in Neurological Diseases, Beijing, China; 7 School of Nursing, Capital Medical University, 100069 Beijing, China

**Keywords:** Deep-vein thrombosis, Intracerebral hemorrhage, Early prophylaxis, Factors, Chinese stroke center alliance

## Abstract

**Background:**

There is substantial evidence to support the use of several methods for preventing deep-vein thrombosis (DVT) following intracerebral hemorrhage (ICH). However, the extent to which these measures are implemented in clinical practice and the factors influencing patients’ receipt of preventive measures remain unclear. Therefore, we aimed to evaluate the rate of the early implementation of DVT prophylaxis and the factors associated with its success in patients with ICH.

**Methods:**

This study enrolled 49,950 patients with spontaneous ICH from the Chinese Stroke Center Alliance (CSCA) between August 2015 and July 2019. Early DVT prophylaxis implementation was defined as an intervention occurring within 48 h after admission. Univariate and multivariate logistic regression analyses were conducted to identify the rate and factors associated with the implementation of early prophylaxis for DVT in patients with ICH.

**Results:**

Among the 49,950 ICH patients, the rate of early DVT prophylaxis implementation was 49.9%, the rate of early mobilization implementation was 29.49%, and that of pharmacological prophylaxis was 2.02%. Factors associated with an increased likelihood of early DVT prophylaxis being administered in the multivariable model included receiving early rehabilitation therapy (odds ratio [OR], 2.531); admission to stroke unit (OR 2.231); admission to intensive care unit (OR 1.975); being located in central (OR 1.879) or eastern regions (OR 1.529); having a history of chronic obstructive pulmonary disease (OR 1.292), ischemic stroke (OR 1.245), coronary heart disease or myocardial infarction (OR 1.2); taking antihypertensive drugs (OR 1.136); and having a higher Glasgow Coma Scale (GCS) score (OR 1.045). Conversely, being male (OR 0.936), being hospitalized in tertiary hospitals (OR 0.778), and having a previous intracranial hemorrhage (OR 0.733) were associated with a lower likelihood of early DVT prophylaxis being administered in patients with ICH.

**Conclusions:**

The implementation rate of early DVT prophylaxis among Chinese patients with ICH was subpar, with pharmacological prophylaxis showing the lowest prevalence. Various controllable factors exerted an impact on the implementation of early DVT prophylaxis in this population.

**Supplementary Information:**

The online version contains supplementary material available at 10.1186/s12959-024-00592-w.

## Background

Deep-vein thrombosis (DVT) is a significant medical condition characterized by the formation of a blood clot within the deep-veins of the body [1]. DVT poses a serious risk, as it can result in pulmonary embolism, a life-threatening condition, and it is a prevalent global health concern [2]. Intracerebral hemorrhage (ICH) is a common and fatal subtype of stroke that has an incidence of 24.6 per 100,000 person-years [3]. It leads to severe neurological impairments and unfavorable functional outcomes in survivors [4]. Research indicates that the prevalence of DVT among ICH patients ranges from 6.3 to 25% [5, 6], surpassing that of individuals with ischemic stroke [7], brain tumor [8], and craniocerebral trauma [9]. During clinical diagnosis and treatment, patients with ICH typically require bed rest for 4–6 weeks while undergoing acute attack management, particularly those who are comatose or have limb dysfunction, leading to reduced limb activity and sluggish blood circulation. Furthermore, ICH patients often exhibit impaired coagulation function, particularly elevated fibrinolytic system activation, heightening the risk of rebleeding. Consequently, the optimal timing for implementing chemical and mechanical prophylactic measures in ICH patients may be delayed to mitigate the risk of rebleeding-related mortality, potentially resulting in increased incidences of lower limb DVT formation [10, 11].

Previous studies have highlighted the occurrence of DVT following ICH, linking it to poor short-term and long-term prognoses and disability in acute ICH patients [12, 13]. Therefore, it is crucial to investigate and mitigate the risk factors associated with DVT in ICH patients. In recent years, a growing body of evidence has provided support for the prevention and management of DVT in ICH patients. Studies have suggested that pharmacological prophylaxis against DVT within 48 h of admission and thrombus pump therapy within 24 h of admission of patients with ICH can effectively reduce the incidence of DVT without significantly increasing the risk of bleeding events [14, 15]. However, the implementation of early prophylaxis measures for DVT in patients with ICH are barely satisfactory. An observational cohort study conducted in the United States revealed that, out of 74,283 patients with cerebral hemorrhage across 1,358 hospitals, only 7.9% received early pharmacological prophylaxis, 89.4% received early mechanical prophylaxis, and 2.6% received no prophylaxis [16]. Similarly, a study conducted in Beijing that collected in-hospital data from 1,355 ICH patients across 15 hospitals found that only 14.2% of ICH patients initially received treatment in a neurological intensive care unit (ICU), and only 22.3% of bedridden patients received preventive DVT treatment within 48 h of symptom onset [17].

In short, the implementation rate of early DVT prophylaxis in patients with ICH varies across academic centers. However, there is currently a lack of large-scale, multi-center studies conducted in China to help clarify the practice of early DVT prophylaxis. Exploring and understanding the implementation rate of early DVT prophylaxis is of utmost importance to nursing practice. Not only does it enable the evaluation of clinical nursing measures, but it also serves as a valuable means to supervise and enhance the overall quality of nursing care. Ultimately, this has the potential to greatly improve patient outcomes. To address this research gap and advance nursing knowledge and practice, conducting rigorous, multi-center studies to investigate the implementation rate of early DVT prophylaxis becomes imperative. In addition, previous research has identified several factors, including female sex; increasing age; comorbidities such as atrial fibrillation, diabetes, coronary issues, prior ischemic stroke, or transient ischemic attack; and hospital characteristics such as geographic location that influence the rate of early DVT prophylaxis among stroke patients [16, 18]. Unfortunately, the results and conclusions of these studies have been inconsistent. Therefore, this study aimed to utilize data from the Chinese Stroke Center Alliance (CSCA) to determine the rate of early DVT prophylaxis administration in ICH patients and identify the potential factors impacting its implementation. The findings will serve as a reference for those promoting nursing practices related to early DVT prophylaxis in this patient population.

## Methods

### Study design

This study utilized data from the CSCA, which was established by the Chinese Stroke Alliance in June 2015 with the aim of enhancing stroke care quality and clinical outcomes. The CSCA was a national, hospital-based, multicentre, voluntary, multifaceted intervention and continuous quality improvement initiative. This programme was made available to all Chinese secondary and tertiary grade hospitals. Patients diagnosed as acute ischemic stroke (AIS), transient ischemic attack, intracerebral hemorrhage, or subarachnoid hemorrhage were enrolled within 7 days of symptom onset and were > 18 years of age. Only in-hospital data were recorded, and patients after discharge were not followed. As of July 2017, 1576 hospitals in China have contributed detailed clinical information to serve as a benchmark for the stroke care quality of 433,264 patients. Data on stroke patients, including general information, disease type, medication history, hospitalization details, outcomes, and complications, are continuously collected and reviewed by trained professionals in accordance with strict protocols. To ensure patient privacy, all data are securely transmitted and de-identified before use. The data collection and application process were overseen by a team of specialists from the National Clinical Research Center for Neurological Disorders, as previously described in detail [19]. This study received institutional review board approval with a consent waiver from the Ethics Committee of Beijing Tiantan Hospital, and written consent was obtained from participating patients.

### Participants

This study enrolled patients with spontaneous ICH from August 2015 to July 2019 who met the inclusion criteria, which included: (1) Age of onset ≥ 18 years old and (2) diagnosis of spontaneous ICH based on the diagnostic criteria formulated in the Chinese Guidelines for Diagnosis and Treatment of ICH 2019 [20], as confirmed by the head of computed tomography examinations and (3) non-ambulatory. Patients were excluded if they had traumatic ICH, ICH caused by aneurysm or vascular malformation, associated cerebral hemorrhage after antithrombolysis or thrombolysis, contraindications for intermittent pneumatic compression (①congestive heart failure, pulmonary edema, and severe lower limb edema; ②deep venous thrombosis, thrombotic phlebitis, or pulmonary embolism; ③abnormalities in the local condition of the lower limbs, vascular lesions, or severe deformities of the lower limbs [21]) or anticoagulants (recent active bleeding and coagulation disorders; severe head or spinal cord injury; platelet count < 20 × 10^9^/L; heparin-induced thrombocytopenia [22]), patient/family refusal, terminal illness/comfort care only, or death before the end of hospital day two.

### Data collection

The outcome indicators encompassed general demographic and clinical characteristics, along with the implementation rate of early DVT prophylaxis in ICH patients across various hospitals and regions.

The data were obtained from the CSCA and included variables such as age, gender, smoking status, Glasgow Coma Scale (GCS), early antithrombotic therapy (antiplatelet or anticoagulation), medical history (ischemic stroke, ICH, subarachnoid hemorrhage, transient ischemic attack [TIA], diabetes mellitus, dyslipidemia, coronary heart disease [CHD] or myocardial infarction [MI], atrial fibrillation, peripheral vascular disease [PVD], chronic heart failure, chronic obstructive pulmonary disease [COPD], and other heart diseases), a history of medication (antithrombotic drugs, antihypertensive drugs, hypoglycemic drugs, or antihyperlipidemic drugs), ward of admission, hospital region, and hospital grade.

Based on guidelines [20], we recommend five types of DVT prophylaxis for patients with ICH: pneumatic compression devices (Essential medical equipment that reduces blood stasis by increasing the peak blood flow velocity through continuous and intermittent pressure methods. They can decrease the likelihood of blood clot formation by alleviating blood stagnation in the veins. Additionally, the application of pressure can exert an antithrombotic effect by enhancing fibrinolytic activity, thereby stimulating the release of plasminogen activator), stockings (graduated compression stockings, thigh-length or knee-length), early mobilization (passive exercises, such as ankle movements and knee flexion and extension exercises, as well as active exercise such as exercises in bed, sitting exercises, bedside standing exercises, balance training, walking, and stair climbing exercises), unfractionated heparin, and low-molecular-weight heparin (LMWH) (enoxaparin sodium, dalteparin sodium, ardeparin sodium, etc.). If any one of these interventions was administered to patients with ICH within 48 h of admission, early DVT prophylaxis was considered to have been implemented and recorded. However, more detailed information regarding the implementation timing, frequency, and specific types of pneumatic compression devices, stockings, and early mobilization was not available.

### Statistical analysis

Baseline characteristics were described as numbers and frequencies for categorical variables and means ± standard deviations (SDs) or medians with interquartile ranges (IQRs) for continuous variables. To compare baseline characteristics between patients who received DVT prophylaxis and those who did not, we used the Average Standard Deviation (ASD) estimator, with the indicator > 10% approximately equivalent to *P* value less than 0.05, indicating a clinically significant imbalance. We assessed the univariate associations between patient or hospital baseline characteristics and DVT prophylaxis using univariate logistic regression models. Subsequently, all statistically significant variables (*P* < 0.05) identified in the univariate logistic analysis were included in a multivariable logistic regression model. To minimize sample size reduction and mitigate bias resulting from non-random missing data, a variable was considered a candidate predictor for the multivariate analysis (using the backward method) if the proportion of missing data in our study was less than 10%.

The relationships between variables and DVT prophylaxis were determined by calculating odds ratios (ORs) with 95% confidence intervals (CIs) via multivariable logistic regression analysis, while adjusting for potential confounding factors. All statistical tests were two-tailed, with *P* < 0.05 considered statistically significant. The analyses were conducted using SAS version 9.4 software (SAS Institute, Cary, NC, USA).

## Results

### Demographic and clinical characteristics of included ICH patients

**Fig. 1:**
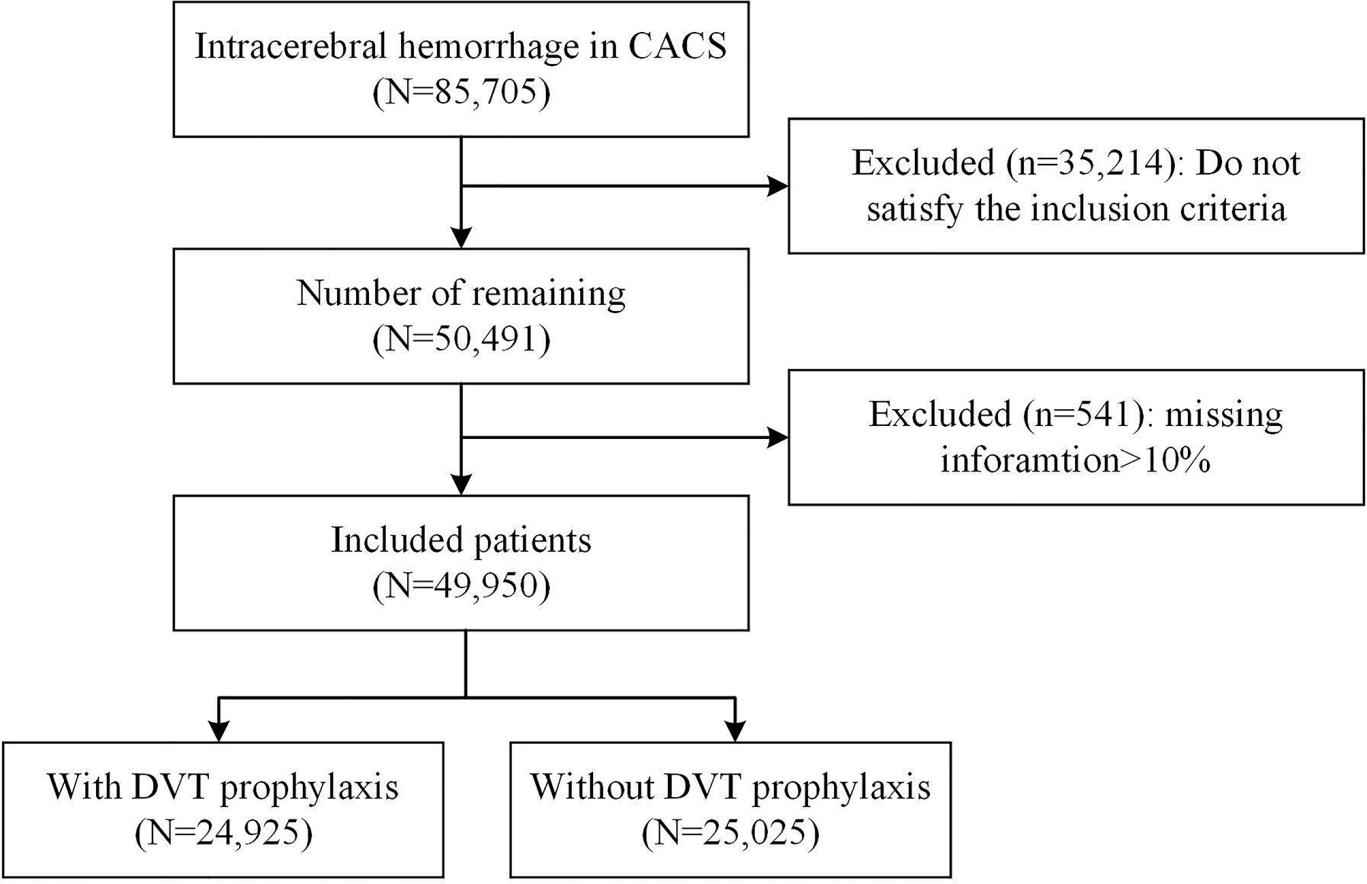
Flow diagram of the participants. This flowchart shows the progress of all participants through the trial. CSCA = Chinese Stroke Center Alliance, DVT = Deep-vein thrombosis

In the CSCA dataset, there were data on 85,705 patients with ICH. Of these, 35,755 cases were excluded because they did not meet the inclusion criteria or their missing data exceeded the 10% cutoff, leaving 49,950 cases for analysis. A flow chart of included subjects is displayed in Fig. 1. The average age of the subjects was 63.1 ± 12.9 years, and there were 19,316 (38.7%) females, and the average GCS score was 10.7 ± 4.1. Among the included patients, 29,129 (58.3%) were from tertiary hospitals, while 20,821 (41.7%) were from secondary hospitals.

We observed that patients without DVT prophylaxis had a lower admission GCS score (mean 10.4, SD 4.2 vs. 11.0, SD 4.0) compared to those with DVT prophylaxis. Additionally, patients without DVT prophylaxis exhibited a lower rate of early rehabilitation therapy (16,566 [66.2%] vs. 20,938 [84.0%], ASD = 42.1%) but a higher rate of ICH history (5,410 [21.6%] vs. 3,915 [15.7%], ASD = 15.2%). Moreover, patients without DVT prophylaxis were less likely to be admitted to stroke units (3,064 [12.2%] vs. 4,466 [17.9%], ASD = 16.0%) or ICUs (5,088 [20.3%] vs. 7,513 [30.1%], ASD = 22.7%), or to be treated in the central regions (10,335 [41.3%] vs. 11,584 [46.5%], ASD = 10.5%). However, they were more likely to be admitted to neurological wards (16,173 [64.6%] vs. 12,588 [50.5%], ASD = 28.8%) and treated in the western regions (6,122 [24.5%] vs. 4,579 [18.4%], ASD = 14.9%). Additional baseline data are presented in Table 1 (see Additional file 1).

### Status of early DVT prophylaxis implementation in patients with ICH

The implementation rate of DVT prophylaxis for patients with ICH was 49.9% (24,925/49,950). Of all the prophylactic measures, early mobilization had the highest implementation rate, at 29.49%, followed by pneumatic compression devices at 21.98%. The implementation rate was lowest for pharmacological prophylaxis measures, at 2.02%. Additional details regarding the implementation of other prophylaxis measures are presented in Table 2 (see Additional file 1).

### Univariate analysis of factors influencing DVT prophylaxis implementation in patients with ICH

Table 3 (see Additional file 1) presents the results of the univariate analysis of factors associated with DVT prophylaxis implementation. The findings revealed significant differences between the groups in terms of age, gender, GCS scores, early rehabilitation therapy, ward of admission, hospital region, hospital grade, history of ischemic stroke, cerebral hemorrhage, TIA, diabetes mellitus, CHD, PVD, other heart diseases, COPD, taking antihypertensive drugs, hypoglycemic drugs, and antihyperlipidemic drugs (all *P* < 0.05).

### Multivariable analysis of factors influencing DVT prophylaxis implementation in patients with ICH

In the multivariable model, factors associated with an increased likelihood of a patient receiving early DVT prophylaxis included receiving early rehabilitation therapy (OR 2.531 [95% CI 2.38–2.69], *P* < 0.001); admission to stroke units (OR 2.231 [95% CI 1.78–2.80], *P* < 0.001); admission to ICUs (OR 1.975 [95% CI 1.58–2.47], *P* < 0.001); being located in central regions (OR 1.879 [95% CI 1.75–2.01], *P* < 0.001) or eastern regions (OR 1.529 [95% CI 1.43–1.64], *P* < 0.001); having a history of COPD (OR 1.292 [95% CI 1.05–1.59], *P* = 0.014), ischemic stroke (OR 1.245 [95% CI 1.15–1.35], *P* < 0.001), CHD or MI (OR 1.2 [95% CI 1.07–1.35], *P* = 0.003); taking antihypertensive drugs (OR 1.136 [95% CI 1.08–1.20], *P* < 0.001); and having a higher GCS score (OR 1.045 [95% CI 1.04–1.05], *P* < 0.001). Conversely, being male (OR 0.936 [95% CI 0.89–0.99], *P* = 0.013), being hospitalized in tertiary hospitals (OR 0.778 [95% CI 0.74–0.82], *P* < 0.001), and having a previous intracranial hemorrhage (OR 0.733 [95% CI 0.69–0.78], *P* < 0.001) were associated with a lower likelihood of patients with ICH receiving early DVT prophylaxis, as shown in Table 4 (see Additional file 1).

## Discussion

This study utilized the CSCA database to analyze the DVT prophylaxis implementation status and factors influencing its administration to Chinese patients with ICH. The study population was representative, as it included patients with ICH from a total of 1,476 hospitals across China. Our findings revealed the significant underutilization of DVT prophylaxis for Chinese patients with ICH, with an implementation rate of only 49.9%. We identified several factors influencing the likelihood of receiving early DVT prophylaxis. Patients who received early rehabilitation therapy; were admitted to stroke units or ICUs; received treatment in central or eastern regions; had a history of COPD, ischemic stroke, CHD or MI; were taking antihypertensive drugs; or had higher GCS scores were more likely to receive early DVT prophylaxis. Conversely, male patients, those hospitalized in tertiary hospitals, and those with a history of intracranial hemorrhage were less likely to be given early DVT prophylaxis.

Implementing early DVT prophylaxis in the acute stage of ICH patient management poses a dilemma because of the need to prevent rebleeding. The 2019 Chinese guidelines for the diagnosis and treatment of acute intracerebral hemorrhage recommend several measures for DVT prevention, including early mobilization, leg elevation, pneumatic compression devices for paralyzed patients, and considering low-dose LMWH or unfractionated heparin for high-risk patients [20]. However, our findings revealed that the overall implementation rate of DVT prophylaxis was low, with non-pharmacological prophylaxis accounting for 58.03% (28,987/49,950), and pharmacological prophylaxis accounting for only 2.02% (1,009/49,950). According to the National Clinical Quality Control Indicators for Critical Care Medicine Survey 2015–2019 [23], the national average proportion of ICU patients who received DVT prophylaxis was 58.24% in 2015 and 59.66% in 2019, which were slightly higher than the results obtained in our study. In addition, this result contrasts with studies conducted in the United States [16], which reported higher rates of early pharmacological prophylaxis (7.9%) and mechanical prophylaxis (89.4%) given to ICH patients. In another retrospective study encompassing 32,690 patients with spontaneous ICH, 5,395 patients (16.5%) received pharmacological prophylaxis, which is also higher than the findings of this study [24]. The substantial disparity between clinical practice and established guidelines in China indicates the need for urgent action to improve compliance with DVT-prevention guidelines. Healthcare professionals often prioritize mechanical prophylactic measures, e.g., early exercise, massage, or pneumatic compression devices, to minimize the risk of rebleeding in ICH patients, leading to the limited implementation of pharmacological prophylaxis for DVT [11]. Addressing the identified barriers, promoting awareness, and fostering adherence to guidelines can play crucial roles in reducing DVT incidence, improving patient outcomes, and minimizing additional financial costs for this vulnerable population [25, 26].

Our study confirmed that patients with ICH who received early rehabilitation therapy were also associated with a higher rate of DVT prophylaxis implementation, which aligns with previous research conducted by Li et al. [18]. Early rehabilitation measures administered by physiotherapists, neuropsychologists, nurses, or speech therapists in the early post-stroke stages typically focus on improving mobility, upper extremity function, language, neglect, and dysphagia [27]. This comprehensive approach enhances patients’ understanding of the risks associated with DVT and the importance of preventive measures, thereby encouraging their active participation in implementing such measures. Additionally, early rehabilitation requires collaborative efforts and meticulous monitoring from the medical team, resulting in increased attention and scrutiny given to patients and subsequently improving the likelihood of DVT prophylaxis implementation.

Furthermore, this study revealed that patients with ICH admitted to ICUs or stroke units had a higher likelihood of receiving DVT prophylaxis. Prolonged stays in ICUs elevate the risk of DVT, prompting medical personnel to intensify DVT prophylaxis measures for patients with ICH in this setting [28]. Supporting this, Li et al.’s study revealed that 98.5% of ICU patients received mechanical or pharmacological prophylaxis measures for DVT [29], while only 59.2% of general surgeons implemented such measures [30]. The stroke unit, a multidisciplinary cooperation model widely used in China for diagnosing and treating acute stroke, provides specialized care and early rehabilitation plans for patients with ICH beyond those provided by conventional wards. These measures increase the likelihood of the early administration of DVT prophylaxis in stroke unit settings [31, 32].

In this study, the implementation rate of DVT prophylaxis for patients with ICH was found to be higher in the central and eastern regions of China compared to the western regions. This discrepancy may be attributed to the relatively greater availability of medical resources and improved medical conditions in the eastern and central regions. Hospitals in these regions have better access to health services, including anticoagulants and pneumatic compression devices, which contribute to the higher implementation rate of DVT prophylaxis measures in the central and eastern regions [18]. Additionally, several patient factors, such as female gender; higher GCS score; a history of ischemic stroke, CHD or MI, COPD; and the use of antihypertensive drugs, were identified as promoting the implementation of DVT prophylaxis. These factors are high-risk indicators for the onset of DVT [9, 28, 33, 34], and their presence raises medical staff’s awareness of the need for DVT prophylaxis in patients with ICH. Various prediction models and evaluation tools for DVT have been established that provide references for the specialized assessment of DVT risk in hospitalized patients [35, 36]. By utilizing these evaluation results, clinical staff can judge the need to implement DVT prophylaxis for high-risk groups, thus increasing the likelihood of DVT prophylaxis being implemented in these populations.

It is worth noting, however, that patients with ICH in tertiary hospitals were found to be less likely to receive DVT prophylaxis, which differs from the findings of Cherian et al. [16]. This difference may be attributed to cultural differences. In China, tertiary hospitals are renowned as high-level medical facilities with over 500 beds. The lower provision of DVT prophylaxis in tertiary hospitals can be attributed to various factors, including complex patient profiles, concerns about developing hemorrhagic complications, and the increased patient demand that may have led to decreased healthcare quality [18]. These factors may influence the decision-making process regarding DVT prophylaxis in these settings. To fully understand the reasons behind the lower implementation rates in tertiary hospitals, it is essential to conduct further research. Future studies should focus on evaluating the specific barriers and challenges faced by tertiary hospitals in providing DVT prophylaxis.

The findings of this study indicate that patients with a history of intracranial hemorrhage are less likely to receive early DVT prophylaxis. This could be attributed to medical professionals cautiously considering the appropriateness of implementing DVT prophylaxis in these patients and weighing up the risks of anticoagulant therapy and hemorrhaging. Nevertheless, several high-level pieces of evidence have demonstrated the effectiveness of pharmacological prophylaxis for DVT in patients with no recurrent bleeding within 24 h and the absence of an increased risk of bleeding [15, 37]. Hence, it is crucial for hospital managers to collaborate and establish a comprehensive management and supervision system for early DVT prophylaxis. This system should prioritize targeted risk assessments, streamlined evaluation procedures and timelines, and efficient diagnostic and treatment processes at both the medical and nursing levels and should aim to strike a balance between minimizing the risk of rebleeding and effectively preventing the occurrence of DVT.

However, there are several limitations to consider. First, retrospective studies inherently have limitations associated with the selection of the patient population. Second, certain important indicators, such as D-dimer, were not included because of the variations in data collection methods across different hospital grades. Third, more detailed information regarding the implementation timing, frequency, and specific types of pneumatic compression devices, stockings, and early mobilization was not available in the current study, which warrant further investigations. Additionally, although most covariates were adjusted for in this study, there may still have been some residual confounding factors that could not be controlled. Lastly, since all patients in the CSCA study were Chinese, which may limit the generalizability of the findings to other populations.

## Conclusions

In conclusion, this study revealed that the implementation rate of DVT prophylaxis in patients with ICH was 49.9%, with early mobilization at 29.49% and pharmacological prophylaxis at 2.02%. Male patients, those hospitalized in tertiary hospitals, and those with a history of intracranial hemorrhage were found to have a lower implementation rate. To improve clinical practice, future research should focus on localizing DVT prevention and treatment guidelines; implementing early warning interventions; and promoting collaboration at national, hospital, and clinical levels for the enhanced evaluation and diagnosis of DVT in ICH patients. These efforts should facilitate effective DVT prophylaxis and reduce DVT occurrence. Overall, this study has provided valuable insights that led to us recommend optimized DVT prevention and management strategies for ICH patients to guide the practice of nursing professionals.

### Electronic supplementary material

Below is the link to the electronic supplementary material.

Additional file 1

## Data Availability

Data is provided within the manuscript or supplementary information files.

## Abbreviations

ASD: Average standard deviation
CHD: Coronary heart disease
CI: Confidence intervals
COPD: Chronic obstructive pulmonary disease
CSCA: Chinese Stroke Center Alliance
DVT: Deep-vein thrombosis
GCS: Glasgow Coma Scale
ICU: Intensive care unit
LMWH: Low-molecular-weight heparin
OR: Odds ratio
SD: Standard deviations
