# Supplementary material for: Implementation of early prophylaxis for deep-vein thrombosis in intracerebral hemorrhage patients: an observational study from the Chinese Stroke Center Alliance
Source: Thromb J. 2024 Feb 28;22:22. doi: 10.1186/s12959-024-00592-w (PMC10900581; doi:10.1186/s12959-024-00592-w)
Supplement: Supplementary file 1 — Additional file 1 [file 12959_2024_592_MOESM1_ESM.docx]

**Table 1.** Baseline data of ICH patients with and without DVT prophylaxis during hospitalization.

| Characteristics | Total (*N*=49,950 [100%]) | With DVT prophylaxis (*N*=24,925 [49.9%]) | Without DVT prophylaxis (*N*=25,025 [50.1%]) | ASD (%) |
| --- | --- | --- | --- | --- |
| Demographics, mean ± SD/ *n* (%) |  | | | |
| Age, year | 63.1±12.9 | 63.2±12.7 | 63.0±13.1 | 1.6 |
| Male | 30,634 (61.3) | 15,133 (60.7) | 15,501 (61.9) | 2.5 |
| Clinical characteristics, mean ± SD/ *n* (%) |  | | | |
| Admission GCS score | 10.7±4.1 | 11.0±4.0 | 10.4±4.2 | 14.6 |
| Early rehabilitation | 37,504 (75.1) | 20,938 (84.0) | 16,566 (66.2) | 42.1 |
| Early antithrombotic therapy | 187 (0.4) | 104 (0.4) | 83 (0.3) | 1.7 |
| Medical history, *n* (%) |  | | | |
| Ischemic stroke | 6,397 (12.8) | 3,461 (13.9) | 2,936 (11.7) | 6.6 |
| ICH | 9,325 (18.7) | 3,915 (15.7) | 5,410 (21.6) | 15.2 |
| SAH | 289 (0.6) | 143 (0.6) | 146 (0.6) | 0.0 |
| TIA | 300 (0.6) | 113 (0.5) | 187 (0.7) | 2.6 |
| Diabetes mellitus | 4,588 (9.2) | 2,389 (9.6) | 2,199 (8.8) | 2.8 |
| Dyslipidemia | 1,976 (4.0) | 839 (3.4) | 1,137 (4.5) | 5.6 |
| CHD or MI | 2,680 (5.4) | 1,492 (6.0) | 1,188 (4.7) | 5.8 |
| AF | 766 (1.5) | 401 (1.6) | 365 (1.5) | 0.8 |

**Table 1.**  Continued

| Characteristics | Total (N=49,950 [100%]) | With DVT prophylaxis (N=24,925 [49.9%]) | Without DVT prophylaxis (N=25,025 [50.1%]) | ASD |
| --- | --- | --- | --- | --- |
| PVD | 464 (0.9) | 203 (0.8) | 261 (1.0) | 2.1 |
| Chronic heart failure | 222 (0.4) | 122 (0.5) | 100 (0.4) | 1.5 |
| COPD | 766 (1.5) | 448 (1.8) | 318 (1.3) | 4.0 |
| Other heart disease | 2,337 (4.7) | 1,297 (5.2) | 1,040 (4.2) | 4.7 |
| Smoking | 16,050 (32.1) | 8,036 (32.2) | 8,014 (32.0) | 0.4 |
| History of medication, *n* (%) |  | | | |
| Antithrombotic drugs | 3,703 (7.4) | 1,905 (7.6) | 1,798 (7.2) | 1.5 |
| Antihypertensive drugs | 23,744 (47.5) | 12,177 (48.9) | 11,567 (46.2) | 5.4 |
| Hypoglycemic drugs | 3,311 (6.6) | 1,726 (6.9) | 1,585 (6.3) | 2.4 |
| Antihyperlipidemic drugs | 2,735 (5.5) | 1,316 (5.3) | 1,419 (5.7) | 1.8 |
| Admitted ward, *n* (%) |  |  |  |  |
| Stroke unit | 7,530 (15.1) | 4,466 (17.9) | 3,064 (12.2) | 16.0 |
| Neurological ward | 28,761 (57.6) | 12,588 (50.5) | 16,173 (64.6) | 28.8 |
| ICU | 12,601 (25.2) | 7,513 (30.1) | 5,088 (20.3) | 22.7 |
| Others ward | 1,058 (2.1) | 358 (1.4) | 700 (2.8) | 9.8 |
| Hospital Region, *n* (%) |  |  |  |  |
| East | 17,330 (34.7) | 8,762 (35.2) | 8,568 (34.2) | 2.1 |
| Center | 21,919 (43.9) | 11,584 (46.5) | 10,335 (41.3) | 10.5 |

**Table 1.**  Continued

| Characteristics | Total (N=49,950 [100%]) | With DVT prophylaxis (N=24,925 [49.9%]) | Without DVT prophylaxis (N=25,025 [50.1%]) | ASD |
| --- | --- | --- | --- | --- |
| West | 10,701 (21.4) | 4,579 (18.4) | 6,122 (24.5) | 14.9 |
| Hospital grade, *n* (%) |  |  |  |  |
| Secondary | 20,821 (41.7) | 10,819 (43.4) | 10,002 (40.0) | 6.9 |
| Tertiary | 29,129 (58.3) | 14,106 (56.6) | 15,023 (60.0) | 6.9 |

Abbreviations: ICH, Intracerebral hemorrhage; DVT, deep-vein thrombosis; SD, standard deviation; GCS, Glasgow coma scale; SAH, subarachnoid hemorrhage; TIA, [Transient ischemic attack](javascript:;); CHD, coronary heart disease; MI, myocardial infarction; AF, atrial fibrillation; PVD, [peripheral vascular disease](javascript:;); COPD, chronic obstructive pulmonary disease; ICU, intensive care unit.

**Table 2.** Type of DVT Prophylaxis in Patients with ICH（%）

| DVT Prophylaxis type | *n* | With DVT prophylaxis (*n*=24925) | Total  (*n*=49,950) |
| --- | --- | --- | --- |
| Pneumatic compression devices | 10,977 | 44.04 | 21.98 |
| Stocking | 3,281 | 13.16 | 6.57 |
| Early mobilization | 14,729 | 59.09 | 29.49 |
| Unfractionated heparin | 143 | 0.57 | 0.29 |
| Low molecular weight heparin | 866 | 3.47 | 1.73 |

Abbreviations: ICH, Intracerebral hemorrhage; DVT, deep-vein thrombosis.

Note: One patient may have combined multiple DVT prophylaxis, and the percentages add up to more than 49.9%; All the above interventions were implemented within 48 hours.

**Table 3.** Univariate analysis of DVT Prophylaxis in patient with ICH

| Variables | OR value | 95% CI | *P* Value |
| --- | --- | --- | --- |
| Demographics |  | | |
| Age | 1.01 | 1.00-1.03 | 0.048 |
| Gender, male | 0.95 | 0.92-0.98 | 0.005 |
| Clinical characteristics |  | | |
| Admission GCS scores | 1.04 | 1.04-1.05 | <0.001 |
| Early rehabilitation | 2.68 | 2.57-2.80 | <0.001 |
| Medical history |  | | |
| Ischemic stroke | 1.21 | 1.15-1.28 | <0.001 |
| Cerebral hemorrhage | 0.68 | 0.65-0.71 | <0.001 |
| SAH | 0.98 | 0.78-1.24 | 0.886 |
| TIA | 0.60 | 0.48-0.76 | <0.001 |
| Diabetes mellitus | 1.10 | 1.04-1.17 | 0.002 |
| Dyslipidemia | 0.73 | 0.67-0.80 | <0.001 |
| CHD/previous MI | 1.28 | 1.18-1.38 | <0.001 |
| AF | 1.10 | 0.96-1.27 | 0.172 |
| PVD | 0.78 | 0.65-0.94 | 0.008 |
| Chronic heart failure | 1.23 | 0.94-1.60 | 0.133 |
| Other heart disease | 1.27 | 1.16-1.38 | <0.001 |
| COPD | 1.42 | 1.23-1.64 | <0.001 |
| Smoking | 1.01 | 0.97-1.05 | 0.604 |
| History of medication |  | | |
| Antithrombotic drugs | 1.07 | 1.00-1.14 | 0.051 |
| Antihypertensive drugs | 1.11 | 1.07-1.15 | <0.001 |
| Hypoglycemic drugs | 1.10 | 1.03-1.18 | 0.008 |
| Antihyperlipidemic drugs | 0.93 | 0.86-1.00 | 0.055 |
| Admitted ward |  |  |  |
| Others | Reference | Reference | Reference |
| Stroke unit | 2.85 | 2.49-3.26 | <0.001 |
| neurological ward | 1.52 | 1.34-1.73 | <0.001 |
| ICU | 2.89 | 2.53-3.30 | <0.001 |
| Hospital Region |  |  |  |
| West | Reference | Reference | Reference |
| East | 1.37 | 1.30-1.44 | <0.001 |
| Central | 1.50 | 1.43-1.57 | <0.001 |
| Hospital grade |  |  |  |
| Secondary | Reference | Reference | Reference |
| Tertiary | 0.87 | 0.84-0.90 | <0.001 |

Abbreviations: DVT, deep-vein thrombosis; ICH, Intracerebral hemorrhage; OR, odd ratio; CI: confidence interval; GCS, Glasgow coma scale; SAH, subarachnoid hemorrhage; TIA, [Transient ischemic attack](javascript:;); CHD, coronary heart disease; MI, myocardial infarction; AF, atrial fibrillation; PVD, [peripheral vascular disease](javascript:;); COPD, chronic obstructive pulmonary disease; ICU, intensive care unit.

**Table 4.** Multivariate analysis of DVT Prophylaxis in patients with ICH

| Variable | OR value | 95% CI | *P* Value |
| --- | --- | --- | --- |
| Gender, Male | 0.936 | 0.89-0.99 | 0.013 |
| Clinical characteristics |  |  |  |
| Early Rehabilitation | 2.531 | 2.38-2.69 | <0.001 |
| Admission GCS scores (Per 1 point) | 1.045 | 1.04-1.05 | <0.001 |
| Medical history |  |  |  |
| Ischemic stroke | 1.245 | 1.15-1.35 | <0.001 |
| Cerebral hemorrhage | 0.733 | 0.69-0.78 | <0.001 |
| CHD or previous MI | 1.200 | 1.07-1.35 | 0.003 |
| COPD | 1.292 | 1.05-1.59 | 0.014 |
| History of medication |  |  |  |
| Antihypertensive agents | 1.136 | 1.08-1.20 | <0.001 |
| Admitted ward |  |  |  |
| Others | Reference | Reference | Reference |
| ICU | 1.975 | 1.58-2.47 | <0.001 |
| Stroke unit | 2.231 | 1.78-2.80 | <0.001 |
| Neurological ward | 1.239 | 0.99-1.54 | 0.056 |
| Hospital Region |  |  |  |
| West | Reference | Reference | Reference |
| East | 1.529 | 1.43-1.64 | <0.001 |
| Central | 1.879 | 1.75-2.01 | <0.001 |
| Hospital grade (Tertiary vs Secondary) | 0.778 | 0.74-0.82 | <0.001 |

Abbreviations: DVT, deep-vein thrombosis; ICH, Intracerebral hemorrhage; OR, odd ratio; CI: confidence interval; GCS, Glasgow coma scale; CHD, coronary heart disease; MI, myocardial infarction; COPD, chronic obstructive pulmonary disease; ICU, intensive care unit.
